# Evaluation of the feasibility of the FAST-M maternal sepsis intervention in Pakistan: a protocol

**DOI:** 10.1186/s40814-022-01090-4

**Published:** 2022-06-24

**Authors:** Sheikh Irfan Ahmed, Raheel Sikandar, Rubina Barolia, Bakhtawar M. Hanif Khowaja, Kashif Ali Memon, James Cheshire, Catherine Dunlop, Arri Coomarasamy, Lumaan Sheikh, David Lissauer

**Affiliations:** 1 Aga Khan University Hospital, National Stadium Road, P.O. Box 3500, Karachi City, 74800 Pakistan; 2 Liaquat University of Health and Medical Sciences, LUMHS Hospital, Hyderabad City, 76090 Pakistan; 3 Institute of Metabolism and Systems Research, University of Birmingham, Edgbaston, Birmingham, B15 2TT UK; 4 Institute of Life Course and Medical Sciences, University of Liverpool, William Henry Duncan Building, Liverpool, L7 8TX UK; 5 Malawi-Liverpool-Wellcome Trust Clinical Research Programme, Chichiri, Blantyre 3, Blantyre, Malawi

**Keywords:** FAST-M intervention, Maternal sepsis, Pakistan, Qualitative study, Sepsis bundle, Care bundle, Complex intervention, Low-resource setting, Feasibility study, Maternal deaths

## Abstract

**Background:**

Maternal sepsis is a life-threatening condition, defined by organ dysfunction caused by infection during pregnancy, childbirth, and the postpartum period. It is estimated to account for between one-tenth and half (4.7% to 13.7%) of all maternal deaths globally. An international stakeholder group, including the World Health Organization, developed a maternal sepsis management bundle called “FAST-M” for resource-limited settings through a synthesis of evidence and international consensus. The FAST-M treatment bundle consists of five components: Fluids, Antibiotics, Source identification and control, assessment of the need to Transport or Transfer to a higher level of care and ongoing Monitoring (of the mother and neonate). This study aims to adapt the FAST-M intervention and evaluate its feasibility in Pakistan.

**Methods:**

The proposed study is a mixed method, with a before and after design. The study will be conducted in two phases at the Liaquat University of Medical and Health Sciences, Hyderabad. In the first phase (formative assessment), we will adapt the bundle care tools for the local context and assess in what circumstances different components of the intervention are likely to be effective, by conducting interviews and a focus group discussion. Qualitative data will be analyzed considering a framework method approach using NVivo version 10 (QSR International, Pty Ltd.) software. The qualitative results will guide the adaptation of FAST-M intervention in local context. In the second phase, we will evaluate the feasibility of the FAST-M intervention. Quantitative analyses will be done to assess numerous outcomes: process, organizational, clinical, structural, and adverse events with quantitative comparisons made before and after implementation of the bundle. Qualitative analysis will be done to evaluate the outcomes of intervention by conducting FGDs with HCPs involved during the implementation process. This will provide an understanding and validation of quantitative findings.

**Discussion:**

The utilization of care bundles can facilitate recognition and timely management of maternal sepsis. There is a need to adapt, integrate, and optimize a bundled care approach in low-resource settings in Pakistan to minimize the burden of maternal morbidities and mortalities due to sepsis.

**Supplementary Information:**

The online version contains supplementary material available at 10.1186/s40814-022-01090-4.

## Background

Pregnancy and childbirth-related complications are a major public health concern [1]. Every day, approximately 830 women die from preventable causes related to pregnancy and childbirth, and almost one-third of these occur in South Asia [2]. Physiological and immunological variations during pregnancy and the postpartum period predispose women to risks of these complications [3]. About 60% of maternal deaths occur during delivery and the postpartum period [4]. The major complications that account for 80% of all maternal deaths include severe bleeding after childbirth, infections (usually after childbirth), hypertension, and unsafe abortion [5].

The World Health Organization estimates suggest that globally, maternal sepsis accounts for about one-tenth of the maternal deaths around the time of childbirth and is the third most common cause of maternal mortality [6, 7]. While the maternal mortality related to sepsis has decreased considerably in high-income countries accounting for 2.1% of the total maternal deaths from the period of 2005 to 2008, the numbers are still high in lower-income countries accounting for up to 15.1% of maternal deaths annually [8]. More recent WHO estimates that were focused specifically on better understanding the contribution of maternal infection to adverse outcomes suggested that up to half of all maternal deaths were actually infection-related [9].

In Pakistan, complications during pregnancy and childbirth are the leading causes of death in women aged 15–45 years, accounting for 20% of all deaths of women of childbearing age [10]. National figures showed that 15% of maternal deaths are reported due to sepsis [11], and maternal sepsis is established as the 3rd leading cause of maternal mortality [12]. Numerous determinants have been identified that increase the susceptibility of women to sepsis, which include shortage of resources, basic infrastructure, availability of antibiotics, lower literacy rate, lower socioeconomic class, lack of antenatal care, and lack of awareness [13].

There are national sepsis guidelines for Pakistan (SGP) which are designed to aid in the recognition and management of sepsis in adults in the local settings and are modelled on the Surviving Sepsis Campaign (SSC) [14]. However, there is still uncertainty about how best to prevent and treat maternal infections and sepsis and how to optimise the implementation of evidence-based practices in low-resource settings.

Whereas a substantial proportion of the improvements in maternal outcomes in high-income countries was attributed to the prevention and appropriate treatment of maternal sepsis [15]. Early warning scores, modules of educational material in routine healthcare settings, and the bundled approach to sepsis management in high-income countries have been effective in reducing maternal mortalities and morbidities [16]. A more rapid completion of a 3-h bundle of sepsis care and rapid administration of antibiotics were found to be associated with lower risk-adjusted in-hospital mortality (*P* < 0.001) [17]. Despite the improvement of sepsis care in high-income countries, there is still a lack of maternal sepsis-care bundle specific to the maternal population of low-resource settings [6].

The development of a maternal sepsis treatment bundle has been identified as an international “Priority Action” [18]. In collaboration with the WHO Maternal Sepsis Initiative, a Delphi approach was adopted to select contributory components to a maternal sepsis treatment bundle in low-resource settings [18]. The components selected were as follows: Fluids, Antibiotics, Source identification and control, assessment of the need to Transport/Transfer to a higher level of care, and ongoing Monitoring (of the mother and neonate). The treatment bundle was named “FAST-M” as a memorable acronym for both communication and awareness raising [18].

The FAST-M intervention was implemented in districts of Malawi to evaluate the feasibility of early identification and management of maternal sepsis and demonstrated significant improvements in maternal sepsis care. The components included a (1) Maternal Early Obstetric Warning Signs (MEOWS) chart and FAST-M decision tool, (2) FAST-M treatment bundle, and (3) The FAST-M implementation program which consisted of the following: training program, sepsis champions, task shifting, performance dashboards, and data feedback to promote systems level change [19].

Following the implementation of the FAST-M intervention, women were more likely to have a complete set of vital signs monitoring compared with the baseline phase (0/163 [0%] versus 169/252 [67.1%], *P* < 0.001). Improvements in sepsis management were seen across all components of the FAST-M treatment bundle, in particular the proportion of women receiving antibiotics within 1 h (3/12 [25.0%] versus 72/107 [67.3%], *P* < 0.004) [19].

The FAST-M intervention has the capacity to strengthen maternal sepsis care as demonstrated in Malawi. We therefore aim to adapt and evaluate the implementation of the FAST-M intervention to assess improvement in maternal sepsis care in the low-resource setting of Pakistan. A mixed-methods approach will be used to plan the intervention and draw study conclusions.

The following are the study objectives:

### Qualitative research objectives

- To adapt FAST-M bundle care tools (MEOWS chart, decision tool, and treatment bundle) to the context of Pakistan
- To understand the barriers and facilitators to these approaches
- To evaluate implementation outcomes of FAST-M intervention in low-resource setting of Pakistan

### Quantitative research objectives

- To assess whether the use of the FAST-M intervention is feasible in the local healthcare system and improves sepsis care (quantitative comparisons made between before and after implementation of the bundle).

### Mixed-methods design

- To integrate quantitative and qualitative findings into the FAST-M bundle care tools for adaptation to the local context
- To evaluate implementation outcomes through quantitative and qualitative findings
- To develop insight and in-depth understanding of the quantitative findings through qualitative follow-up with healthcare providers

## Methods and analysis

### Study aim

This study aims to determine whether it is feasible to introduce a complex intervention (including a bundled approach) for maternal sepsis care in the low-resource setting of Pakistan.

### Study design

A sequential mixed-method intervention design will be used. This two-phase design will start with the qualitative phase to collect data for the adaptation of FAST-M bundle care tools and will be applied to make these tools contextual based. In the first qualitative phase (formative assessment), we will identify healthcare providers and health officials for key informant interviews and the focus group discussion. The healthcare providers and the officials working at the study site with experience in managing sepsis patients will be selected. These individuals will be interviewed to explore ideas and views that will guide the intervention phase.

Data collected from the qualitative phase will be utilized to adapt bundle care tools and plan training programs. This will be then followed by the implementation of contextual-based modified FAST-M tools in the study setting and will allow us to assess the practicality of implementation.

The implementation outcomes will be evaluated using both the quantitative and qualitative methods (summative evaluation) to validate the study findings. Quantitative comparisons will be drawn before and after the implementation to distinguish study outcomes. Focus group discussions with healthcare providers and officials will provide in-depth qualitative findings regarding implementation outcomes.

### Rationale for design

The mixed-methods approach involves qualitative and quantitative data collection, analysis, and integration within the same study to provide a better and more comprehensive answer to research questions [19, 20]. A sequential mixed-method intervention model will be used to gain insights into the existing local practices in order to adapt the FAST-M intervention and evaluate implementation outcomes of a locally adapted intervention.

The FAST-M intervention is new in Pakistan, and its feasibility assessment will require adaptation before implementing it at the study setting. Thus, this study will begin with qualitative and quantitative assessments to understand the existing practices and culture of maternal sepsis care to modify bundle care tools in the context of local setting. This will support the study team to plan and provide useful and practical approach for the intervention. Baseline facility audit tool (quantitative observational data through CRF) will determine existing resources and practices to manage maternal sepsis, resource availability, and infection control infrastructure in the facility, whereas the qualitative approach will explore the views of healthcare providers and their readiness for the intervention, potential challenges, and enablers for implementation.

While evaluating the implementation outcomes, qualitative and quantitative data will be combined to validate the study findings. The quantitative data will provide findings on patients’ outcomes through the collection of observational data, and the qualitative findings will help in validating quantitative findings. It will also explore the HCPs’ readiness, facilitators, challenges, and reflection on the implementation. Therefore, the feasibility of this complex intervention will be assessed using mixed methods to gather evidence through qualitative and quantitative approaches and confirm our study findings.

**Fig. 1:**
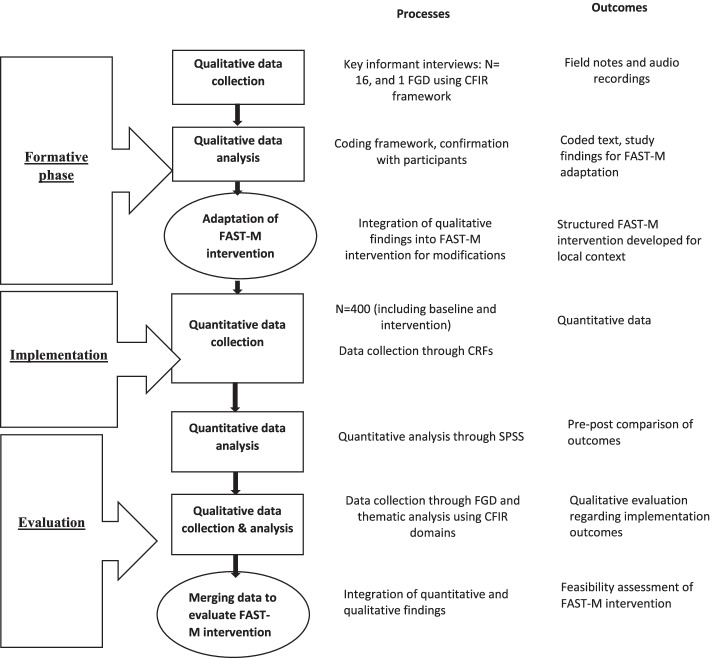
Overview and flow of study using qualitative and quantitative strands

Figure 1 provides the overview and flow of study using qualitative and quantitative strands.

### Study setting

The study will be conducted at the Liaquat University of Medical Health Sciences (LUMHS), which is a public sector tertiary hospital located in the Hyderabad district of Pakistan. The hospital has a total of 3000 beds and 35 departments which serve a large number of mostly underprivileged populations. The hospital provides 24-h emergency cover to patients coming from nearby urban and rural areas. LUMHS has three obstetrics and gynecology units.

The current data from the facility shows that a total of approximately 11,205 patients were admitted to OBGYN units from the period of January to August 2021, and 210/11,205 (1.87%) women were diagnosed with maternal sepsis. The maternal mortality rate in these units was recorded as 159/11205 (1.4%). Out of these 159 deaths, 45 were due to confirmed maternal sepsis (28.3%). These indicators demonstrate that there is a need for a robust system to early detect and manage maternal sepsis cases in the hospital.

### Study procedures

#### Adaptation phase

For a FAST-M bundle to be effective in Pakistan, it is necessary to identify how best to implement this approach in the context of local setting. In order to adapt this intervention, a systematic method will be taken to understand the nature of existing practices and an appropriate system for characterizing the intervention and its components that can make use of this understanding. This constitutes phase 1 of the study.

This formative research (phase 1) will adopt a qualitative research design involving focus group discussion (FGD) and key informant interviews (KIIs) and a purposive sampling approach. The aim of group discussion and interviews will be to engage health practitioners, government officials, and other key stakeholders to understand the behavior of existing practices in the study setting for maternal sepsis care, to finalize the FAST-M tools for the context of Pakistan, and to identify various facilitators and barriers that may influence the implementation of the FAST-M intervention. The FGD and KIIs will be conducted using interview guides developed through the use of the Consolidated Framework for Implementation Research (CFIR) [21].

#### Consolidated Framework for Implementation Research (CFIR)

The CFIR is a commonly used framework to facilitate the implementation of research design, evaluation, and implement evidence-based interventions. It comprises five major domains: (1) intervention characteristics, (2) outer setting, (3) inner setting, (4) characteristics of individuals, and (5) process of implementation. CFIR is categorized as a determinant framework with the objective to understand and explain factors (individual or organization) that influence implementation outcomes [21]. This framework has been used in a wide range of studies because this flexible framework can be tailored to different settings across multiple contexts [22]. We aim to use the tailored CFIR framework to assess critical barriers and facilitators to implementation that need to be addressed at multiple levels if the FAST-M bundle is to be successfully optimized and integrated in healthcare practices in Pakistan (Supplementary file 1).

The interview guides (see Supplementary file 1) for KIIs and the FGD have been developed using five major domains of CFIR to identify existing practices for sepsis management. These guides contain questions that will help identify the facilitators and barriers to the implementation of FAST-M intervention in the study setting. The identification of existing practices for maternal sepsis care and facilitators and barriers in phase 1 will then form the basis of adaptation of tools in local context for feasibility testing of FAST-M intervention in phase 2.

#### Sampling

Key informant interviews and the FGD will be conducted with the following:

- HCPs including physicians, nursing staff, and healthcare administrators who are associated with maternal sepsis care and management
- HCPs who have worked at the study site for the last 6 months

Fifteen to 20 semi-structured key informant interviews and a focus group discussion are planned in the qualitative phase of the study until data saturation is reached [23].

#### Data collection

Semi-structured interview guides have been developed to explore healthcare professionals’ views and attitudes towards FAST-M intervention and its implementation at their facility. Before beginning the interview, the qualitative researchers will describe the FAST-M bundle components and the patient referral pathway demonstrating the algorithm and summary for utilization of FAST-M bundle care tools (Supplementary file 2).

A free flow of discussion among participants will be encouraged, using probes from these discussions to obtain healthcare professionals’ perceptions about the feasibility of the FAST-M intervention. Interviews will be conducted face to face in Urdu and English according to the participants’ preference and will be audio recorded following consent from study participants. Interviews and focus group discussions will be conducted by experienced study team members who are also trained, qualitative researchers. Detailed field notes will be also taken during each interview to capture nonverbal language and cues.

All data will be kept confidential for 7 years on password-protected computers and/or locked filing cabinets only accessible to members of the research team. During transcription, audio recordings will be referenced only with an identification number for anonymity of participants, with all identifying information removed before using the software analysis tool.

#### COVID-19 standard operating procedures (SOPs)

In view of the current COVID-19 pandemic situation, all project-related activities will comply with standard operating procedures (SOPs). The following measures will be taken related to this study: (1) all research staff will be provided with appropriate masks, sanitizers, and/or other applicable personal-protective equipment (PPE) to the field staff; (2) daily mandatory screening for COVID-19 symptoms of all project staff; and (3) KIIs and FGDs will be conducted with social distancing (6 feet) with all participants wearing face coverings.

#### Data analysis

Qualitative data gained during formative assessment through individual interviews and FGDs in phase 1 will be audio-recorded, transcribed, and analyzed using content analysis approach and the inductive analysis methods to determine the facilitators and barriers for implementation of the intervention and will be summarized according to CFIR domains. This will help to understand the important contextual features that are helping or hindering the operationalization of the FAST-M intervention.

The codes, categories, and themes will be developed using NVivo version 10 (QSR International, Pty Ltd.) software [24]. The primary team will review the codes and associated themes multiple times to check for potential biases, to ensure they are reflecting participants’ words and meanings, and to improve the credibility of interpretation of the interviews. Initial findings will be shared with a group of participants to help with interpretation and generate meaning from the data. The FAST-M bundle care tools (MEOWS chart, decision tool, and treatment bundle) will be modified through construal gained from interviews and discussions with healthcare providers.

#### Baseline phase

Following phase 1, a baseline assessment phase of 2 months will be carried out to assess existing resources and practices to prevent and treat maternal sepsis. The case report form (CRF) will be used for assessing baseline resource availability, practitioner’s knowledge, and infection control infrastructure. Based on the findings from baseline assessments, necessary infrastructure and training to establish FAST-M decision intervention will be introduced to the study site. The baseline data will later be used to compare if the introduction of the intervention affects clinical practice. This will enable us to compare whether the subsequent introduction of the intervention affects existing practice.

#### Training program

Multiple full day training sessions by the study team will be delivered to healthcare practitioners working for maternal care and sepsis management at the study site. The interactive sessions will be offered in English and Urdu languages for each healthcare practitioner to understand the practices completely. Any requirement for supplementary educational material such as posters and a study booklet will be determined via feedback from frontline clinical staff and stakeholders on facilitators and barriers to the use of these tools. This will be done using qualitative interviews and focus groups discussion during phase 1.

The training and implementation program is likely to consist of the following:

- Background information on maternal sepsis, including risk factors, signs and symptoms, and the potential consequences if untreated
- Use of the MEOWS chart to track and trigger the recognition of deteriorating patients
- Use of the FAST-M decision tool to recognize and screen for potential study participants at risk of maternal sepsis
- Use of the FAST-M treatment tool to initiate the bundle components
- Guidance around implementing the individual components of the FAST-M bundle
- Use of feedback tools (run chart and dashboard) and approaches the team can use to work together to improve compliance and outcomes

Post-training, an impact survey will be made to measure the extent to which skills and knowledge learned in the program has translated into improved behavior among participants who attended the training program.

#### Intervention phase

At the start of the intervention phase, FAST-M bundle care tools (Additional file 2) will be introduced including MEOWS chart, FAST-M decision tool, and FAST-M treatment bundle.

**Fig. 2:**
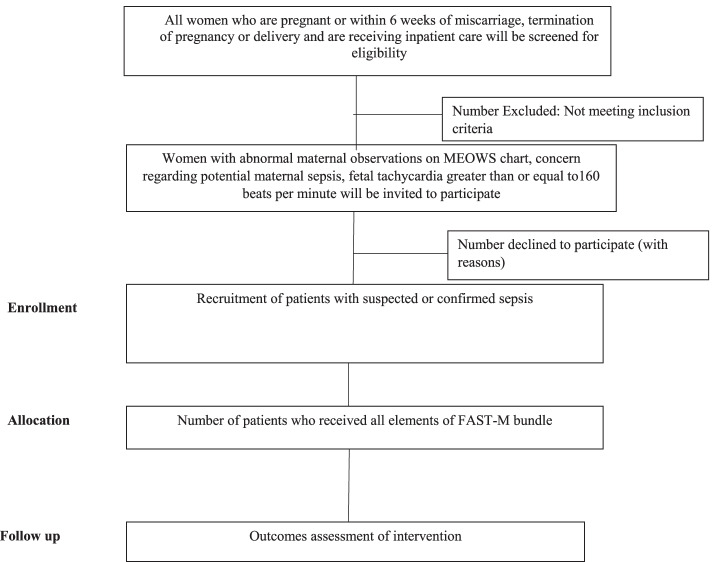
Study period

Figure 2 provides the summary of assessment, enrollment, and intervention.

#### Modified Early Obstetric Warning Score

MEOWS stands for Modified Early Obstetric Warning Score (MEOWS) to identify suspected maternal sepsis patients. This tool helps in identifying any early warning scores used to track the physiological parameters of an individual over time onto a chart, with guidance thresholds to trigger clinical action if they become abnormal [25]. The MEOWS chart used during the implementation of the FAST-M intervention in the districts of Malawi will be adapted in the context of Pakistan for the purpose of this feasibility study [19].

The use of obstetric early warning systems (OEWS) in UK maternity units was recommended in the 2007 Confidential Enquiry into Maternal and Child Health (CEMACH) report as an adjunct to reducing maternal morbidity and mortality [25]. MEOWS included in the 2007 UK Confidential Enquiry consisted of scores of respiratory rate, oxygen saturation, temperature, heart rate, blood pressure, assessment of urine, including for proteinuria, color of amniotic fluid, neurological response, pain score, assessment of lochia, and an overall assessment of whether the woman appears well [25]. Clinical action is triggered by a single parameter exceeding a red threshold or any two parameters exceeding a yellow threshold. MEOWs chart has been widely adopted in the UK and internationally [26].

To complete the MEOWS chart, the healthcare providers involved in the study will be trained to record patient observations (heart rate, respiratory rate, blood pressure, conscious level, urine output, and temperature) and fetal heart rate (if applicable) from medical records. These observations will be charted on a MEOWS chart in the inpatient setting.

#### Decision tool

Abnormal observations (indicated by a single red or two yellow thresholds) will trigger a review by an attending doctor or nurse. This will be agreed locally prior to study commencement. These patients will then be screened for potential sepsis using the FAST-M decision tool. In addition to abnormal maternal observations, cases of suspected sepsis will also be identified using the FAST-M patient pathway when prompted by attending clinician concern regarding potential maternal infection or an increased fetal heart rate greater than or equal to160 beats per minute (in pregnant women).

Patients will be defined as having or are at a higher risk of having sepsis, who will trigger a red flag on the decision tool and will be commenced immediately on the FAST-M treatment bundle pathway. These patients will receive a review from a doctor/nurse as soon as possible, with the bundle initiated within 1 h. Those patients who trigger two yellow flags on the decision tool and have or are at a higher risk of having sepsis require a review from a doctor/nurse within 3 h. All suspected cases will remain in observation for possible development of red flags. Half-hourly (if red triggers) or hourly (if two yellows trigger), observations will be made in the first instance until otherwise specified by an attending clinical decision-maker. Those patients without at least one red or two yellow flags will be considered to have a low risk of sepsis and will be managed according to local guidelines by the screening healthcare practitioner.

#### FAST-M treatment bundle

Patients managed with the FAST-M treatment bundle will have their treatment recorded on the FAST-M treatment bundle form including documentation of actions completed and any reasons for not completing certain components of the bundle.

The FAST-M treatment bundle consists of the timely consideration of all the following:

- Fluids
- Antibiotics
- Source identification and control
- Assessment of the need to transport/transfer to a high level of care
- Ongoing monitoring (of the mother and neonate)

#### Sampling

During the intervention phase, patients will be assessed by a healthcare practitioner on the decision to initiate screening for potential maternal sepsis that will be based on the following inclusion criteria:

- Women who are pregnant or within 6 weeks of miscarriage, termination of pregnancy, or delivery
- Abnormal maternal observations triggered on the inpatient MEOWS chart
- Healthcare practitioner’s concern regarding potential maternal sepsis
- In pregnant women, fetal tachycardia greater than or equal to160 beats per minute

For enrollment of sepsis cases, we will power to a primary process outcome of “sepsis management compliance”. This is defined as “the proportion of patients admitted with features of sepsis who receive appropriate monitoring (full set of vital sign measurements on admission) and antibiotics within 1 h (if required)”. This means the notes of all patients with suspected or confirmed sepsis will be reviewed, and their data would be collected using study case report forms (CRFs).

**Fig. 3:**
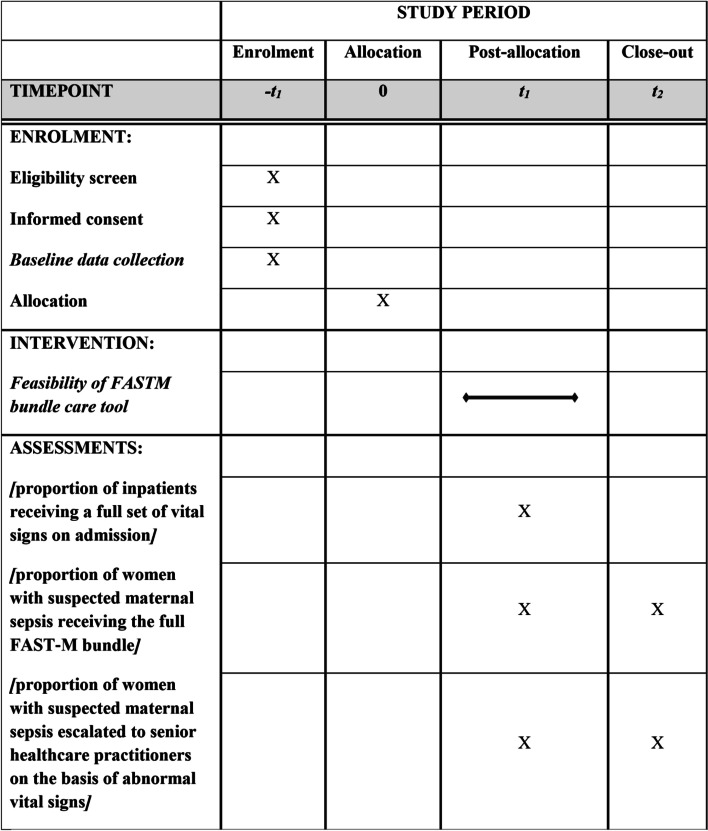
Flow of participants through the study

Assuming baseline compliance is less than 10%, to detect an increase in compliance to 20%, with an alpha of 0.05, we will require the observation of 199 participants in each phase (baseline and intervention) to achieve a power of 80%. The test statistics used is 1-tailed test. This is adequate precision to allow important increases to be estimated. Allowing for loss to follow-up and missing laboratory results, we will take 210 in each phase, as appropriate to allow the study to have adequate power to detect an increase in compliance. This number of cases will be feasible to collect within 6 months, based on the current rate of sepsis from hospital records of the anticipated site. The flow of participants through the study is presented in Fig. 3.

#### Data collection

During the intervention phase, data will be collected by a member of the research team who will not be part of the clinical team. Data will be collected using CRFs on various outcomes: structural, clinical, organizational, and any adverse events.

If the patient requires a transfer as part of the FAST-M treatment bundle to any other health facility due to shortage of beds or other resources, the data collector will continue to follow up on the patient’s clinical outcomes. The data collection team will keep their study site updated on their performance using this data and will visually display it on run charts and dashboards and work on strategies to improve performance. The data will be maintained in an investigator file to be secured in a locked cabinet. The information recorded on the data collection sheet will be recorded in a database located on a secure server. Quantitative data (data collection on CRFs) will be measured every 2 weeks throughout the intervention phase.

#### Data analysis

Quantitative analyses will be done to assess numerous outcomes: process, organizational, clinical, structural, and adverse events with quantitative comparisons made before and after implementation of the bundle. Quantitative data will be analyzed using percentages, means, medians interquartile ranges and 95% confidence intervals, and the change identified over time.

### Co-interventions for implementation of the intervention

#### Clinical champions

The local clinical champions and team leaders will be identified during training programs. Healthcare practitioners who have been involved in the management of patients with maternal sepsis or infections for at least 6 months and have demonstrated capacity and enthusiasm will be recruited as champions for maternal sepsis intervention as well as key members of the study management group. These champions will be trained to take a lead from different units at the study site and will remain engaged throughout the implementation process. The overarching goal of each champion will be to encourage engagement and compliance with the FAST-M bundle. To achieve this goal, champions at each site will be engaged in a number of key activities: disseminating knowledge, advocating, navigating boundaries, facilitating consensus, arranging meetings with stakeholders, tracking quality indicators, and developing organizational communication strategies and relationships.

#### Ongoing improvement approaches

Ongoing improvement practices at different units of the study site will be carried out by clinical champions of the respective units. The improvement strategies include the following: (1) weekly/biweekly training of healthcare providers on FAST-M tools; (2) display of run charts, dashboards in units to demonstrate the rate of maternal sepsis and outcomes of maternal sepsis cases over time; and (3) meeting with stakeholders for communicating needs and requirements for implementation of the FAST-M intervention. Table 1 shows the summary of ongoing improvement approaches planned to implement for FAST-M implementation.

**Table 1:** Summary of FAST-M implementation approach

Approaches	Planned strategies
Facility-level approaches	Site leadership by project champion Formation of local sepsis committee Formal site launch
Individual-level approaches	Multidisciplinary, scenario-based local training Coaching by local project champion Aide-memoires, posters Paper-based tools (MEOWS chart, decision tool, treatment tool) Task sharing of vital sign measurement
Ongoing improvement approaches	Site-based performance dashboards and run charts Local problem-solving: led by sepsis committee (ongoing quality improvement, ownership, local adaptations, engagement, learning climate, and sustainability)

#### Evaluation phase

We will explore a range of outcomes measurement through quantitative data for maternal sepsis care. Primary process includes (1) the proportion of patients admitted with features of sepsis who received appropriate monitoring (full set of vital sign measurements on admission recorded on MEOWS chart), (2) the proportion of women with suspected maternal sepsis received antibiotics within 1 h (if required), and (3) the proportion of women with suspected maternal sepsis receiving the FAST-M treatment bundle (including each bundle component) within 1 h of identification of sepsis. Secondary outcomes will include the following: (1) the proportion of women with suspected maternal sepsis referred to clinical decision-maker on the basis of abnormal vital signs records and (2) the proportion of women with suspected maternal sepsis receiving a clinical review by a senior clinical decision-maker following their diagnosis.

For qualitative evaluation, two to three follow-up focus group discussions will be conducted with HCPs involved during the process of implementation of the FAST-M intervention. The HCPs will be invited for group discussions from all participating units to share their views and perceptions regarding implementation outcomes. This will provide an opportunity to gain more insights into quantitative findings and explore the observational results in more depth. The interview guides (see Supplementary file 1) for FGDs have been developed using five major domains of CFIR to evaluate the outcomes and feasibility of the FAST-M intervention. These discussions will be audio recorded following consent from study participants. Focus group discussions will be conducted by experienced study team members who are also trained, qualitative researchers. Detailed field notes will be also taken during discussions to capture nonverbal language and cues.

Qualitative data gained through FGDs will be transcribed and analyzed using thematic analysis approach and the deductive analysis methods. The CFIR domains will be used as themes and constructs as subthemes for the purpose of analysis [21]. The research team will conduct multiple reviews of the transcripts and tapes to familiarize themselves with the data that will be reflexive and interactive. Analysis will begin soon after first FGD is completed and will be continued concurrently with data collection to help determine when new information is no longer being generated. Although we identified the CFIR as the appropriate framework, additional codes may emerge during the familiarization process to develop from the experiences of participants.

An audit trail will be used to document our decision-making process. The codes, subthemes, and themes will be developed using NVivo version 10 (QSR International, Pty Ltd.) software [24]. The primary team will review the codes and associated themes multiple times to check for potential biases, to ensure they are reflecting participants’ words and meanings, and to improve the credibility of the interpretation of the interviews.

#### Data integration

The mixed-methods design of the study using both the qualitative and quantitative data will be utilized throughout the study to implement the intervention, determine outcome results, and validate the study findings. This approach will support in explaining and building upon quantitative results through qualitative findings and will provide in-depth data to justify significant study outcomes.

The integration of data will occur at two points. First, when we will adapt the FAST-M intervention by means of combining baseline quantitative data (facility audit tool) findings and qualitative findings to create contextual-based adapted tools. The qualitative data will explore views and perceptions of HCPs to understand observational records. The study team will use the findings from the initial exploratory databases to build into a feature that can be analyzed quantitatively. The development of locally adapted tools will base on both quantitative and qualitative data and will increase the content validity of the study.

Second, when reporting evaluation outcomes using qualitative and quantitative results through a joint display. The overall process of implementation and modifications in the inner or outer setting before and after the implementation will be determined through observational findings and personal feedback. The results of this mixed-methods study will be displayed through the CFIR framework. The follow-up on the experiences of HCPs after implementation will help in using actual participant quotes and matching them with the quantitative survey results to explain the study outcomes.

**Fig. 4:**
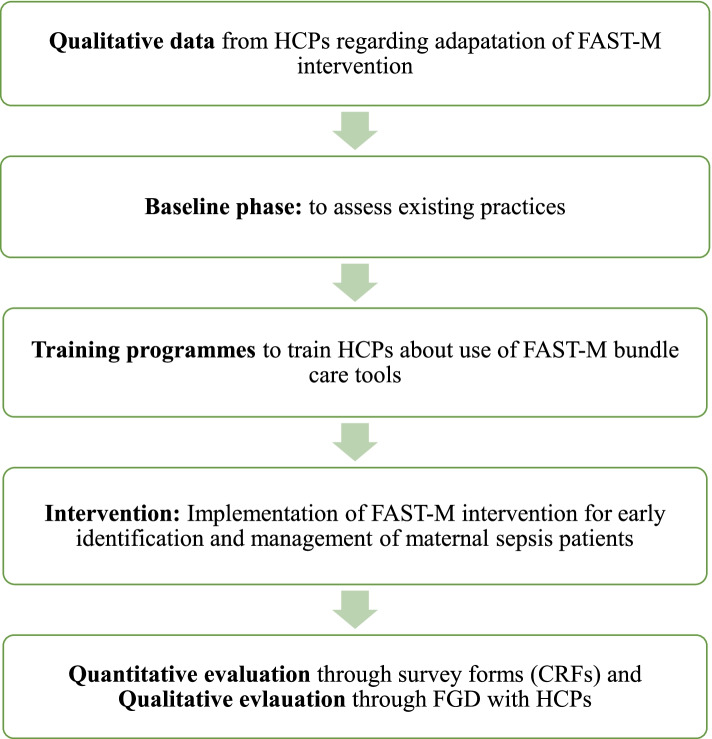
Overview of study implementation

Figure 4 displays the complete overview of study procedures and implementation.

#### Potential harms

Fluid resuscitation in patients with sepsis if not managed appropriately can precipitate volume overload and subsequent pulmonary edema. This is a particular concern in patients with preeclampsia. Clear teaching and guidance regarding fluid resuscitation will be provided during the training programs. The participants will be evaluated regarding their understanding of fluid administration through a post-test. When fluid resuscitating patients with suspected maternal sepsis, the decision regarding the rate of fluid administration will be made by the responsible clinician based on clinical examination findings and ongoing monitoring and will be documented in patients’ files. The data for fluid administration will be collected through a review of patients’ records, and all information related to its indication, rate, and duration will be recorded on study CRFs.

## Discussion

Overall, bundle care tools have the potential to enhance improvements in sepsis care [6]. However, the implementation challenges posed by these bundles should be examined, especially in low-resource settings.

The FAST-M maternal sepsis intervention has the potential to be used as an integrated strategy for early recognition and management of maternal sepsis in the low-resource health settings.

This mixed-method study will establish whether it is feasible to implement the FAST-M bundle for early identification and management of maternal sepsis in Pakistan. The long-term vision is that the intervention will be trialled in other settings across Pakistan. A large multicountry interventional trial is also anticipated to ascertain the effectiveness of the bundle to improve maternal sepsis care and outcomes in other low- and middle-income countries. The study findings will be disseminated to clinicians and key stakeholders to formulate appropriate bundle care tools for sepsis care. This will help reduce the high rate of maternal mortalities caused by sepsis.

## Supplementary Information

**Additional file 1: Supplementary file 1.** Interview Guide

**Additional file 2: Supplementary file 2.** Study guide for focus group (Evaluation of implementation)

**Additional file 3: Supplementary file 3.** Good Reporting of a Mixed Methods Study (GRAMMS) checklist Guideline Section

## Data Availability

All data developed for this intervention is available from the corresponding author on reasonable request.

## Abbreviations

CFIR: Consolidated Framework for Implementation Research
FAST-M: Fluids, Antibiotics, Source control, assessment of the need to Transport/Transfer to a higher level of care and ongoing Monitoring (of the mother and neonate)
FGD: Focus group discussion
HCPs: Healthcare providers
KIIs: Key informant interviews
LMIC: Low- and middle-income countries
LUMHS: Liaquat University of Medical Health Sciences
MEOWS: Maternal Early Obstetric Warning Signs
SSC: Surviving Sepsis Campaign
