# Supplementary material for: Evaluation of the feasibility of the FAST-M maternal sepsis intervention in Pakistan: a protocol
Source: Pilot Feasibility Stud. 2022 Jun 24;8:130. doi: 10.1186/s40814-022-01090-4 (PMC9229426; doi:10.1186/s40814-022-01090-4)
Supplement: Supplementary file 1 — Additional file 1: Supplementary file 1. Interview Guide [file 40814_2022_1090_MOESM1_ESM.pdf]

Interview Guide

Intervention Characteristics

1. What do you know about the intervention or its implementation?
2. How different is this intervention from your existing practices?
3. What kind of information or evidence are you aware of that shows whether or not the intervention will work in your setting?
4. What kinds of changes or alterations do you think you will need to make to the intervention so it will work effectively in your setting?
   - Do you think you will be able to make these changes? Why or why not?
5. What is your perception of the bundling of the intervention for implementation and quality of the supporting materials? Prompts: format, design, user-friendly. Duration, scope, intricacy and number of steps

Outer Setting

6. How do you think the individuals served by your organization will respond to the intervention?
7. What barriers will the individuals served by your organization face to participating in the intervention?
8. What kind of local, state, or national performance measures, policies, regulations, or guidelines might be important in influencing how this intervention can be implemented?

Inner Setting

9. Can you describe how the intervention will be integrated into current processes?
10. What are your current guidelines to assess and manage patients with maternal sepsis? Probes: tool, framework or guidelines for maternal sepsis, lactate test
11. What is your knowledge about importance of lactate test and what is your current practice about lactate testing? Probes: implications for lactate test, guidelines for lactate test
12. What is your current patient to doctor and patient to nurse’s ratio in your setting?

13. Explain the role of doctors and nurses in management of maternal sepsis in your organization. Which cadre is responsible for care and at what level of care? Probes: nurses, doctors, technicians and other health care cadres
14. Other than human resources, what resources are utilized in management of maternal sepsis in your hospital?
15. Do you expect to have sufficient resources to implement and administer the intervention?
    - [If no] What resources will not be available? Probes: human resource, equipments, critical units etc
16. Do you feel the training planned for you will prepare you to carry out the roles and responsibilities expected of you?
    - What are the positive aspects of planned training? What is missing?

Characteristics of Individuals

17. How do you feel about the intervention being used in your setting?
18. Do you think the intervention will be effective in your setting? Why or why not?

Process

19. Who will lead implementation of the intervention?
20. Are there people in your organization who are likely to champion (go above and beyond what might be expected) the intervention?
    Prompts: Position of these champions have in your organization?
21. How do you think they will help with implementation?
