# Supplementary material for: Evaluation of the feasibility of the FAST-M maternal sepsis intervention in Pakistan: a protocol
Source: Pilot Feasibility Stud. 2022 Jun 24;8:130. doi: 10.1186/s40814-022-01090-4 (PMC9229426; doi:10.1186/s40814-022-01090-4)
Supplement: Supplementary file 2 — Additional file 2: Supplementary file 2. Study guide for focus group (Evaluation of implementation) [file 40814_2022_1090_MOESM2_ESM.pdf]

Study guide for focus group (Evaluation of implementation)

Thank you so much for reading the participant information sheet and consenting to participate in our research project.

Questions:

Let's start the discussion by talking about the FAST-M intervention and its implementation in your study setting?

How different was this intervention from your existing practices and how was this integrated into your process?
Probes: charts, communication, systematized plan for monitoring, patient documentation, records

What kind of information or evidence showed whether or not the intervention worked in your setting?
Probes: statistics, transfer of patients, sepsis rates, maternal morbidity, and mortality, administration of bundle components

Did you have sufficient resources to implement and administer the intervention?
Probes: Fluids, antibiotics, laboratory, ambulances, stretchers, FAST-M tools, monitoring equipment, human resource

Who were the key individuals who implemented the intervention?
Probes: doctors, interns, Hos, nurses, residents, admin, HODs

Do you feel the champions selected, helped in the implementation of the FAST-M intervention?

Probes: in communication, in arranging supplies and equipment, in coordination with stakeholders, in implementation of FAST-M intervention

Do you think the intervention was effective in your setting? Why or why not?
Probes: Patient improvement, length of stay of patients, patient transfer

What are your views about the sustainability of this intervention in your setting?
Probes: sepsis policies, protocols, integration of FAST-M intervention

Do you think this intervention helped to improve maternal and newborn health outcomes?
Probes: length of stay, fetal and neonatal wellbeing, transfer of newborn to pediatric critical units, condition of mothers and newborns at discharge, follow-up status of patients

What are your views about using this intervention to improve patient outcomes?
Probes: early detection of infection, deterioration of health, transfer of patients, timely monitoring

What strategies and activities helped you in implementation?
Probes: the display of posters, clinical champions, refreshers, training, monitoring visits, coordination with HODs

What were the barriers to the implementation of intervention? And what actions were taken to remove those barriers?
Probes: resources, the rigidity of HCPs to change the existing policies, compliance, administrative and clinical support

What are your views about using the MEOWs chart for early detection of maternal sepsis cases?
Probes: red triggers, yellow triggers, pre-eclamptic cases, patients condition, frequent monitoring

How effective was the decision tool in making decisions about sepsis patients?
Probes: clinical review, timely administration of treatment bundle

What are your views about the applicability of the overall elements of the FAST-M bundle that is Fluids, Antibiotics, Source control, Transport, and monitoring for management of maternal sepsis cases?
Probes: timely administration, need for administration, reasons for not administring, availability of resources

That concludes our focus group. Thank you so much for coming and sharing your thoughts and opinions with us. We have a short evaluation form that we would like you to fill out if you have time. If you have additional information that you did not get to say in the focus group, please feel free to write it on this evaluation form.
