# Supplementary material for: Evaluation of the feasibility of the FAST-M maternal sepsis intervention in Pakistan: a protocol
Source: Pilot Feasibility Stud. 2022 Jun 24;8:130. doi: 10.1186/s40814-022-01090-4 (PMC9229426; doi:10.1186/s40814-022-01090-4)
Supplement: Supplementary file 3 — Additional file 3: Supplementary file 3. Good Reporting of a Mixed Methods Study (GRAMMS) checklist Guideline Section [file 40814_2022_1090_MOESM3_ESM.pdf]

Good Reporting of a Mixed Methods Study (GRAMMS) checklist Guideline Section

Guideline	Section: page
Describe the justification for using a mixed methods approach to the research question	Section: Rationale for design<br>Pages: 10-11
Describe the design in terms of the purpose, priority and sequence of methods	Section: study design<br>Pages: 9 to 10
Describe each method in terms of sampling, data collection and analysis	Section: Study Procedures<br>Pages 12-25
Describe where integration has occurred, how it has occurred and who has participated in it	Section: Data Integration<br>Pages: 25-26
Describe any limitation of one method associated with the present of the other method	Not Applicable
Describe any insights gained from mixing or integrating methods	Section: Data integration<br>Pages: 25-26
